# Zearalenone and Its Metabolites in Blood Serum, Urine, and Milk of Dairy Cows

**DOI:** 10.3390/ani12131651

**Published:** 2022-06-27

**Authors:** Rimvydas Falkauskas, Bronius Bakutis, Jurgita Jovaišienė, Gintarė Vaičiulienė, Gediminas Gerulis, Sigita Kerzienė, Ingrida Jacevičienė, Eugenijus Jacevičius, Violeta Baliukonienė

**Affiliations:** 1 Department of Food Safety and Quality, Faculty of Veterinary, Lithuanian University of Health Sciences, Tilzes Str. 18, LT-47181 Kaunas, Lithuania; bronius.bakutis@lsmuni.lt (B.B.); jurgita.jovaisiene@lsmuni.lt (J.J.); gintare.vaiciuliene@lsmuni.lt (G.V.); gediminas.gerulis@lsmuni.lt (G.G.); violeta.baliukoniene@lsmuni.lt (V.B.); 2 Department of Animal Breeding, Faculty of Animal Science, Lithuanian University of Health Sciences, Tilzes Str. 18, LT-47181 Kaunas, Lithuania; sigita.kerziene@lsmuni.lt; 3 National Food and Veterinary Risk Assessment Institute, J. Kairiukscio Str. 10, LT-08409 Vilnius, Lithuania; ingrida.jaceviciene@nmvrvi.lt (I.J.); eugenijus.jacevicius@nmvrvi.lt (E.J.)

**Keywords:** zearalenone, zearalanone, cows, β-derivatives, α-derivatives

## Abstract

**Simple Summary:**

The metabolism of zearalenone in dairy cows may affect the manifestation of adverse effects (such as decreased productivity, resistance to pathogens, and estrogenic effects). The aim of this study was to determine how the concentrations of zearalenone metabolites in the blood serum and urine of dairy cows changed when the cows were fed TMR with naturally contaminated ZEN for two weeks, followed by two weeks of feeding with TMR without mycotoxins. In addition, we evaluated the correlation between zearalenone and its metabolites in different biological fluids and feeds.

**Abstract:**

After oral contamination, zearalenone (ZEN) is rapidly absorbed in organisms and can be detected in biological fluids. In this study, we investigated the metabolites of ZEN in the biological fluids of cows (blood, urine, milk). The study was divided into three stages: preparation (the first stage), investigation (the second stage), and final stage (the third stage). Samples of biological fluids were taken 7, 21, and 35 days after the beginning of the study. At the first stage and at the second stage, the cows were fed a total mixed ration (TMR) with naturally contaminated mycotoxin-zearalenone (500 ± 75 µg/kg). In the third stage, the cows were fed a TMR without mycotoxins. This study established that at the second stage, the alpha-zearalenol concentrations in the serum increased by 92% and the beta-zearalanol in the urine decreased by 48% compared to the first stage (*p* < 0.05). The beta-zearalenol and zearalanone concentrations in the urine were higher compared to that of the alpha-zearalenol. The zearalenone concentration in the milk at the second stage was 35% higher than at the first stage (*p* < 0.05). A significant negative correlation (r = –0.540) was determined between the beta-zearalenol and beta-zearalanol concentrations in the urine and the positive significant correlation (r = 0.826) between the beta-zearalanol and alpha-zearalenol concentrations in the serum (*p* < 0.05). During the study, it was determined that feeding cows for two weeks with a TMR without mycotoxins can reduce concentrations of alpha-zearalanol, beta-zearalenol, and beta-zearalanol in the biological fluids and can reduce the concentrations of ZEN in the milk, but does not reduce the concentration of zearalanone.

## 1. Introduction

Mycotoxins are structurally diverse, low-molecular-weight, fungal secondary metabolites that are harmful at low concentrations to farm animals and humans [1,2]. Zearalenone (ZEN) is one of the approximately 300 to 400 mycotoxins that have been identified. ZEN is frequently reported as a mycotoxin due to its safety concerns and economic impact [3,4]. ZEN is one of the most important *Fusarium* spp. mycotoxins, and it is produced by several species, including *F. graminearum*, *F. culmorum*, *F. cerealis*, and *F. equiseti* [2]. ZEN is thermostable, and is not degraded by processing, such as milling, extrusion, storage, or heating [5,6]. ZEN is one of the three most abundant mycotoxins in complete animal feed. Continued fungal growth and ZEN synthesis might develop under poor storage conditions. ZEN is an estrogenic fusariotoxin; it is classified as a phytoestrogen, or as a mycoestrogen. Its chemical structure, which analogous to those of natural estrogens, permits its binding with estrogenic receptor sites, leading to amplified estrogenicity [7]. As a result, ZEN intoxication most often leads to disorders of the reproductive system [8].

ZEN is mainly metabolized at the intestinal and hepatic level and transformed into several metabolites by hydroxylation, glucuronidation, or conjugation reactions [8,9]. The biotransformation of ZEN leads primarily to the formation of two metabolites, alpha-zearalenol (α-ZEL) and beta-zearalenol (β-ZEL), which can be further reduced to alpha-zearalanol (α-ZAL) and beta-zearalanol (β-ZAL) [10]. According to Mahato et al. [7]’s review, α-ZAL is predominantly metabolized into β-ZAL and, to a lesser extent, into zearalanone (ZAN), but Metzler et al. [11] indicate that ZAN can be directly metabolized from ZEN. Other researchers claimed that the major metabolites known to have affinities with estrogenic receptors are in the following order: α-ZAL > α-ZEL > β-ZAL > ZEN > β-ZEL or α-ZEL > α-ZAL > ZEN ≈ ZAN ≈ β-ZAL > β-ZEL [12]. The European Food Safety Authority (EFSA)’s opinion is that α -ZEL is 60 times as potent and estrogenic as ZEN, whereas β-ZEL is only 0.2 times as potent as ZEN [13]. According to Liu and Applegate [14], cows are more resistant to the estrogenic effect of ZEN because they bio-transform ZEN more into β-ZEL than into α-ZEL. ZEN metabolites are found in various animal tissues (the liver, intestines, etc.) and fluids, including blood, urine, and milk [14].

In dairy cows, the risk of exposure to ZEN can be assessed directly, by identifying mycotoxins in the feed, or indirectly, by analyzing the respective biomarkers in the biological fluids. The initial decomposition of mycotoxins begins in the rumen of a cow; the rumen microbiota is one of the factors necessary for the process to take place. Therefore, the concentration of ZEN and its metabolites can be more reliably quantified in blood, urine, and milk samples [15,16,17].

This study hypothesized that the ZEN metabolite concentration in biological fluids depends on the feeding time and the ZEN concentration in the TMR. The aim of the study was to determine the difference between the zearalenone metabolite concentrations in the blood serum and urine of dairy cows, and the concentrations of zearalenone in milk when cows are fed TMR with mycotoxins and later fed TMR without mycotoxins. Furthermore, the study evaluated the correlation between zearalenone and its metabolites in different biological fluids and feed.

## 2. Materials and Methods

### 2.1. Experimental Animals, Feed, and Design

The study was carried out on a dairy cow farm in 2020. Ten clinically healthy Lithuanian Holstein black and white cows between the second and the third lactation with milk yield of 25–35 L/day were selected. The cows were kept in free housing and fed a total mixed ration (TMR) with ZEN—500 ± 75 µg/kg (in dry matter (DM)) twice daily, at 6:00 and 18:00 h. Aflatoxin B1 (AFB1), aflatoxin B2 (AFB2), aflatoxin G1 (AFG1), aflatoxin G2 (AFG2), ochratoxin A (OTA), deoxynivalenol (DON) T-2 toxin, and HT-2 toxin concentrations in feed were below the limit of detection (LOD).

ZEN concentration in TMR was selected by the maximum limit (ML) allowed in dairy cows’ feed by the Lithuanian legal act (ML in feed—500 µg/kg). In order to maintain the same concentration of ZEN in the TMR, corn silage was used.

TMR was formulated to meet or exceed the requirements of a 550-kilogram Holstein cow producing 35 kg/d of milk. The average assessment of the body condition of cows on the five-point scale was 3.0 ± 0.10. TMR for cows is composed of 45% grass silage, 20% corn silage, 15% grass hay, 15% grain concentrate mash (50% barely and 50% wheat), and 5% mineral mixture. The ration was composed of a dry-matter (DM) (%) value of 47.40, acid detergent fiber (% of DM) value of 19.40, neutral detergent fiber (% of DM) value of 28.40, non-fibrous carbohydrate (% of DM) value of 37.20, crude protein (% of DM) value of 14.90, and net energy-for-lactation value of 1.5 (Mcal/kg).

The study was divided into three stages. The preparation stage, at the beginning of the study, continued for 7 days (the first stage). Cows were fed TMR with naturally contaminated mycotoxin ZEN—500 ± 75 µg/kg (in DM). The investigation stage lasted for 14 days, between 8 and 21 day (the second stage); ZEN concentration in TMR was the same as in the first stage. During the final stage, which also lasted 14 days, between days 22 and 35 (the third stage), the cows were fed TMR without mycotoxins or mycotoxin concentrations below LOD. Samples of blood, urine, and milk were taken 7, 21, and 35 days after the beginning of the study.

### 2.2. Samples

Blood samples (about 8 mL) were collected from the left jugular vein using vacutainer tubes without anticoagulant (BD Vacutainer^®^, Plymouth, UK) after milking. The collected blood samples were stored in an ice bath until all samples were taken. The samples were allowed to settle for about 1.5 h before centrifuging at 3000 rpm and 6 °C for 30 min to obtain serum, which was then frozen at −25 ± 3 °C for subsequent analyses. The concentrations of zearalenone metabolites in serum samples were analyzed according to the 2.4 paragraph.

Urine samples (about 60 mL) were taken into 100-milliliter sterile plastic containers by massaging below the vulva. Samples were taken in the morning before feeding the animals. The samples were stored at a temperature of −18 ± 2 °C until testing. The concentrations of zearalenone metabolites in urine samples were analyzed according to the 2.4 paragraph.

Milk samples (about 8 mL) were collected in a tube containing a preservative based on boric acid (Merck KGaA, Darmstadt, Germany). Before taking the milk sample, each teat was cleaned with a tissue moistened with 70% ethanol. The first two streams of milk were discharged, after which milk samples were collected in a tube containing a preservative to study ZEN. The samples were stored at a temperature of −18 ± 2 °C until testing. The concentrations of zearalenone in milk samples were analyzed according to 2.5 paragraph.

### 2.3. Determination of Mycotoxin Concentrations in Feed by HPLC

Contamination with aflatoxin B_1_ (AFB_1_), aflatoxin B_2_ (AFB_2_), aflatoxin G_1_ (AFG_1_), aflatoxin G_2_ (AFG_2_), zearalenone (ZEN), ochratoxin A (OTA), T-2 toxin, and HT-2 toxin in TMR samples was tested by high-performance liquid chromatography (HPLC) with a fluorescent detector (FLD) (model LCMS-8060, Shimadzu Corp., Kyoto, Japan) and deoxynivalenol (DON) by HPLC with mass spectrometry and an ultraviolet detector (UV) (Model SciexAPI 5000, McKinley Scientific, NJ, USA).

The samples were air-dried, ground to pass through a 1-millimeter sieve, and homogenized. Extraction of samples was performed in distilled water for DON, AFB_1_, AFB_2_, AFG_1_, AFG_2_, ZEN, in acetonitrile (ACN)/deionized water (H_2_O) (75:25 *v*/*v*) for T-2 toxin, HT-2 toxin, and OTA in ACN/H_2_O (60:40 *v*/*v*) under orbital shaker (RS–OS 10/20, Phoenix Instrument GmbH, Garbsen, Germany) for 60 min at 23 °C. After extraction, feed samples were centrifuged at a relative centrifugal force (RCF) of 3468 rpm for 10 min (Centrifuge MPW-251, MPW, Warsaw, Poland). Subsequently, they were filtered with PTFE syringe filters through 0.22-micrometer-diameter pores (Millex-GS, Millipore, Billerica, MA, USA) and diluted with phosphate buffered saline (PBS). Next, for the sample-purification step, the extracts were passed over a multi-mycotoxin immunoaffinity column 11 + Myco MS-PREP^®^, (R–Biopharm AG, Pfungstadt, Germany) according to the manufacturer’s recommendations. For determining T-2 toxin and HT-2 toxin, derivatization of TMR samples was performed according to the method described by Kachuei et al. [18], with some modifications.

Chromatographic mycotoxin separation was achieved using a LiChrospher^®^ 100 RP-18, LiChroCART 250-4 column (250 × 4.0 mm, 5 µm; Supelco Park, Bellefonte, PA, USA). Mycotoxins were determined by comparing peak retention fold with standard solutions. 

HPLC conditions for mycotoxins were as follows. Column temperature for AFB_1_, AFB_2_, AFG_1_, AFG_2_, ZEN, OTA and DON: 30 °C. For T-2 toxin and HT-2 toxin: 40 °C. Mobile faze for ZEN and OTA: H_2_O/ACN/ methanol (MeOH) (46/46/8 *v*/*v*/*v*). For DON: H_2_O/ACN/MeOH (94/3/3 *v*/*v*/*v*). For AFB_1_, AFB_2_, AFG_1_, AFG_2_: H_2_O/ACN/MeOH (60/20/30 *v*/*v*/*v*). For T-2 toxin and HT-2 toxin: H_2_O/ACN (40/60 *v*/*v*). Flow rate in mL/min for all mycotoxins: 1 mL/min. Injection volume for all mycotoxins: 100 μL. Fluorescent detector, wavelength λ (nm) (excitation and emission) for AFB_1_, AFB_2_, AFG_1_, AFG_2_: 365 and 435. For T-2 toxin and HT-2 toxin: 381 and 470. For ZEN: 274 and 418. For OTA: 333 and 460. UV detector λ (nm) for DON: 218. LOD: AFB_1_, AFB_2_, AFG_1_, AFG_2_, and OTA—0.2 μg/kg. ZEN—3 μg/kg. DON—20 μg/kg. T-2 toxin and HT-2 toxin—1.4 μg/kg.

### 2.4. Determination of Alpha-Zearalenole, Alpha-Zearalanole, Beta-Zearalanole, Beta-Zearalenole, and Zearalanone Concentrations in Blood-Serum and Urine Samples by GC-MS EI

Zearalenone metabolites in blood and urine samples were tested by gas chromatograph with a mass spectrometer and automatic sample-entry system by applying the electron-mode method (GC-MS EI) (Agilent Technologies 6890N with automatic sample-entry system 5975, Agilent, Santa Clara, CA, USA). The test was performed in a few steps: sample hydrolysis using Helix Pomatia with tert-butylmethylether, purification using Chromabond^®^ C_18_ (Macherey-Nagel GmbH & Co. KG, Düren, Germany) and Chromabond^®^ NH_2_ (Macherey-Nagel GmbH & Co. KG, Düren, Germany) columns for solid phase extraction. Metabolites were determined by adding 2 mL 2 mol/L acetate buffer and 25 μL Helix pomatia and 100 μL internal standard solution, mixing and performing hydrolysis. After hydrolysis, 5 mL tert-butylmethylether was added and after 5 min, the sample was centrifuged for 10 min at 3500 rpm (Centrifuge MPW—251, MPW, Warsaw, Poland). The extract was evaporated to a dry residue with a stream of nitrogen. The dry residue was dissolved in 3 mL MeOH and 1 mL 0.04 mol/L acetate buffer, 2 mL n-hexane was added, and the mixture was centrifuged. After adding 5 mL dichloromethane, centrifuging was continued (Centrifuge MPW—251, MPW, Poland). After 20 min, the sample was evaporated using stream of nitrogen. The obtained dry residue was dissolved in 5 mL of MeOH/H_2_O (40/60, *v*/*v*). The extract was passed through a 500-milligram C_18_ column. The column was washed with MeOH/H_2_O (40/60, *v*/*v*) and acetone/water (20/80, *v*/*v*) and dried under weak vacuum conditions. The residue was dissolved in acetone/methanol (80/20, *v*/*v*), passed through the NH_2_ column and evaporated to dry residue. GC-MS EI analysis parameters are presented in Table 1. 

### 2.5. Determination of Zearalenone in Milk by ELISA

The zearalenone (ZEN) in milk was tested by the enzyme-linked immunosorbent assay (ELISA) method. The RIDASCREEN^®^ Zearalenon (Art. No.: R1401) quantitative test kit (R-Biopharm AG, Darmstadt, Germany) was used for the analysis. Zearalenone extraction was performed according to the manufacturer’s instructions. Milk samples were centrifuged at 3.000 rpm, at 4 °C, for 15 min (Centrifuge MPW—251, MPW, Warsaw, Poland). The upper cream layers were removed. Twenty μL of glucuronidase/arylsulphatase Helix pomatia were added to one mL of a sample, and incubated for 3 h at 37 °C. After incubation, 0.1 mL of MeOH was added to 0.9 mL of hydrolyzed and defatted milk and 50 μL of the resulting solution was used in the assay. Standards were prepared freshly each test day in skimmed milk containing 10% MeOH. The ELISA procedure was carried out based on the manufacturer’s instructions. Absorbance was determined using a microtiter plate spectrophotometer (Bio-tek Synergy HT, Garden Grove, CA, USA) at 450 nm. LOD was approx. 0.06 µg/L.

### 2.6. Statistical Analysis

The statistical analysis of data was performed using the IBM SPSS 25.0 statistical package (SPSS Inc., Chicago, IL, USA). The normality of data was checked using Kolmogorov–Smirnov Test with Lilliefors significance correction. The test showed a normal distribution of the majority of investigated parameters. The statistical significance of the differences between stages was evaluated by parametric Student’s t-test for pair samples. However, since the compared samples were small, differences were also assessed by the non-parametric Wilcoxon signed ranks test, but the results did not differ from the results of the parametric analysis. Test results were compatible. The correlation between zearalenone and its metabolites in blood and urine and zearalenone in feed and milk was assessed using the Pearson correlation. The differences were considered statistically significant when *p* < 0.05.

## 3. Results

### 3.1. Concentrations of Zearalenone Metabolites in Blood-Serum and Urine Samples from Dairy Cows

The detection percentages of the ZEN metabolites in the blood serum of the dairy cows were different. No α-ZAL were detected in the tested samples of blood serum, or their concentrations were below LOD. Meanwhile, β-ZEL and ZAN were detected in all the study stages. It was determined that the α-ZEL concentration in the blood serum at the end of the first and the second stages were statistically significant (*p* < 0.05) (Table 2). The study results also showed that the α-ZEL concentration in the blood serum at the end of the second stage was 14 times (*p* < 0.05) higher than at the end of the first stage. At the end of the third stage, the α-ZEL concentration was twice as low as at the end of the second stage (*p* > 0.05). The highest concentrations of α-ZEL and β-ZEL were at the end of the second stage.

The tested samples of urine also showed variations in the percentage of ZEN metabolites. In these samples, the β-ZAL concentration at the end of the second stage was statistically significant (*p* < 0.05) compared with the first and the third stages (Table 3). It was determined that the ZAN concentrations in the urine samples at the end of the second stage were 22% higher than at the end of the first stage (*p* < 0.05). At the end of the third stage, the β-ZEL and α-ZAL concentrations decreased by 11% and 2.8%, respectively, compared with the end of the second stage. The ZAN and β-ZAL concentrations in the urine samples at the end of the third stage were 35% and 39% higher, respectively, than at the end of the second stage.

### 3.2. Zearalenone Concentrations in Dairy Cows’ Milk Samples

**Figure 1:**
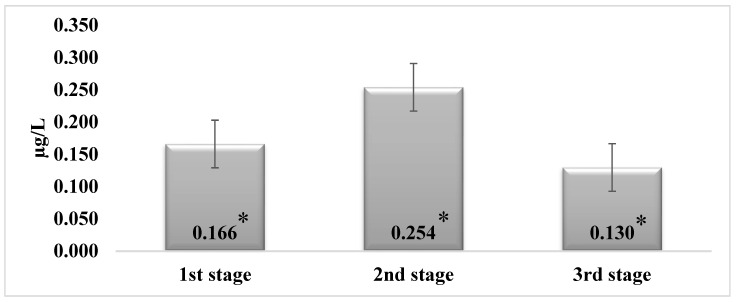
Concentration of zearalenone in dairy cows’ milk samples by stages. * *p* < 0.05.

Zearalenone was detected in 60% of the tested milk samples (the first stage—60%, the second stage—80%, the third stage—40%). It was determined that the ZEN concentrations in the milk samples at the end of the second stage were 34.6% higher than at the end of the first stage (*p* < 0.05). When the cows were fed TMR with mycotoxin concentrations in the third stage, the ZEN concentration in the milk samples decreased by 48.8% (*p* < 0.05) (Figure 1).

### 3.3. Correlation between ZEN Metabolites in Blood-Serum and Urine Samples and Zearalenone in Feed and Milk

In this study, the correlation between the zearalenone metabolites in the blood serum and urine and the zearalenone in the feed and milk of dairy cows was evaluated (Table 4). It was determined that the β-ZAL in the serum samples had a strong positive correlation with the α-ZEL in the blood serum (*p* < 0.05). A moderate positive correlation was found between the β-ZEL and the ZAN in the blood-serum samples (*p* < 0.05). The ZAN in the urine showed a moderate positive correlation with the ZAN in the blood-serum samples (*p* < 0.05). A moderate negative correlation was found between the β-ZEL and the β-ZAL in the urine samples (*p* < 0.05). A strong positive correlation was found between the β-ZAL and the α-ZAL in the urine with the ZEN in the feed (*p* < 0.05). A weak positive correlation was determined between the ZEN in the feed and the ZEN in the milk (*p* > 0.05).

## 4. Discussion

The aims of this study were to determine and evaluate the changes in the ZEN metabolites in the blood serum and urine of dairy cows, determine the concentration of zearalenone in the milk samples, and evaluate the correlation between zearalenone and its metabolites in different biological fluids and feeds. The first stage was designed to avoid possible stress on the dairy cows due to changes in the mycotoxin concentrations in their feed and is regarded as the beginning of the study. The second stage was designed to determine whether the concentrations of the ZEN metabolites in the organism increased. The third stage aimed to answer the question of whether discontinuing the feeding of the TMR with ZEN for two weeks can reduce the concentrations of ZEN metabolites in dairy cows’ blood serum and urine and the ZEN concentration in their milk.

According to Rogowska et al. [19] and Dänicke et al. [20], zearalenone rapidly passes from the digestive tract of ruminants into the organism. In the current study, it was determined that already at the end of the first stage, α-ZEL, β-ZEL, and ZAN concentrations were already detected in the samples of blood serum. At the end of the first study stage, not only ZEN metabolites, which were in the blood serum samples, but also other ZEN derivates, such as α-ZAL and β-ZAL, were detected in the urine samples. The obtained results differed from those published by Polish scientists [15], who maintained that α-ZEL and β-ZEL concentrations either were absent from the blood serum of cows or occurred in amounts below LOD.

In this study, ZEN metabolites were detected. It was found that the β-ZEL concentrations in the blood serum and urine samples after the end of the first stage and at the end of the third stage were higher than the α-ZEL concentrations. This might have been due to endogenic detoxification processes in the liver and rumen of the dairy cows, through which ZEN was converted β-ZEL, which is less toxic than α-ZEL. The obtained results only partially confirm Liu and Applegate [14]’s position that the α-ZEL concentration in the blood serum of ruminants is higher than those of other ZEN metabolites. The results obtained by Dänicke et al. [21] showed that the α-ZEL concentrations in the organisms of dairy cows are lower than those of other ZEN metabolites. In our study, the lowest α-ZEL concentration was determined only in the urine samples, while in the serum, the α-ZEL concentration was not lower than the concentrations of the other ZEN metabolites. According to Malekinejad et al. [22] and Zhang et al. [23], β-ZEL is the main and most common metabolite detected in cows. Our results support this position. Mahato et al. [7], in their review, stated that α-ZAL is metabolized into β-ZAL more often than into ZAN. The results in this study are not consistent with this position, because the ZAN concentrations in the urine samples throughout the study were twice as high as those of the β-ZAL. The ZAN concentrations in the blood-serum samples were 20 to 40 times higher than those of the β-ZAL, depending on the study stage. Summarizing the results obtained in this study regarding the concentrations of ZEN metabolites in the dairy cows, we agree with the European Food Safety Authority (EFSA) [24] that β-derivatives are more common in the blood serum and urine of dairy cows than α-derivatives. In the EFSA’s opinion, α-derivatives are more common in pigs and turkeys.

Previous studies have shown that the duration of the feed intake may affect the concentration of ZEN in milk. German scientists found that for seven consecutive days of feeding cows TMR with a ZEN content of 200 mg/day, the ZEN concentration in milk samples reached 0.7% of the daily ZEN content [25], while a study in China found that raw milk had an average ZEN concentration of 0.015 μg/L [26]. In this study, it was found that when feeding cows TMR with ZEN after the first stage, the ZEN concentration was 0.03% in milk, while after the second stage, it was 0.05%, and after the third stage, it was 0.02% compared with the ZEN concentration in the TMR. The average ZEN concentration in the milk was 0.18 μg/L. These ZEN data increased until the end of the second stage and support Seeling et al. [27]’s position that the continual feeding of ZEN to cows may increase the ZEN in their biological fluids due to the organism’s tendency to accumulate toxins in its tissues and biological fluids. The ZEN levels determined in the milk shows that the organism does not remove all the ZEN it consumes through urine and feces, and part of the ZEN enters the final human-consumption product—milk. Based on a statistically significant correlation, it was found that increases in the ZAN concentration in blood serum may be influenced by the ZAN concentration in urine, while the increased ZEN concentration in feed may be influenced by the α-ZAL concentration in urine and influenced by the ZEN concentration in milk. These correlations in biological fluids confirmed that increased ZEN concentrations in feed can influence the ZEN and its metabolites in biological fluids.

## 5. Conclusions

The results of this study suggest that feeding cows with feed contaminated with ZEN, requiring endogenous detoxification in the rumen, can produce ZEN metabolites, which are distributed in different concentrations in the biological fluids. In this study, the concentrations of α-ZEL in the blood serum and β-ZAL in the urine samples depended on the ZEN concentration in the feed. It was determined that β-derivatives are more common than α-derivatives in biological fluids. In the present study, the highest concentration of ZAN was found in the serum and the highest concentration of β-ZEL was found in the urine. Moreover, this study also suggests that since not all ZEN are metabolized into metabolites, some of the ZEN can be transmitted to raw milk, thereby potentially contaminating milk products. In order to evaluate the concentration of ZEN contamination in milk, further investigations are necessary.

## Figures and Tables

**Table 1:** Gas chromatograph with mass spectrometer and automatic sample-entry system analysis parameters.

Parameters	Zearalenone Metabolites				
	α-ZEL	α-ZAL	β-ZEL	β-ZAL	ZAN
Stationary-phase layer (μm)	0.25	0.25	0.25	0.25	0.25
Injection, in splitless regime (μL)	1	1	1	1	1
Temperature of automatic sample-entry system (°C)	260	260	260	260	260
Temperature of MS ion source/temperature of MS quadrupole (°C)	230/150	230/150	230/150	230/150	230/150
Line velocity of stream speed (cm/s)	46	46	46	46	46
Limit of detection (LOD), (μg/L)	0.04	0.04	0.05	0.05	0.04

α-ZEL, alpha-zearalenol; β-ZEL, beta-zearalenol; ZAN, zearalanone; α-ZAL, alpha-zearalanol; β-ZAL, beta-zearalanol.

**Table 2:** Concentrations of ZEN metabolites in blood-serum samples of dairy cows by stages.

		Zearalenone Metabolites				
		α-ZEL	α-ZAL	β-ZEL	β-ZAL	ZAN
1st stage (*n* = 10)	n (pos)	60	0	100	0	100
	min (µg/L)	0.145	<0.04	1.232	<0.05	1.133
	max (µg/L)	0.348	<0.04	1.964	<0.05	4.257
	mean (µg/L)	0.144 ^a^	<0.04	1.577	<0.05	2.88
2nd stage (*n* = 10)	n (pos)	100	0	100	60	100
	min (µg/L)	0.566	<0.04	0.337	0.095	2.116
	max (µg/L)	3.267	<0.04	2.327	0.366	5.858
	mean (µg/L)	2.028 ^b^	<0.04	1.714	0.154	3.581
3rd stage (*n* = 10)	n (pos)	80	0	100	40	100
	min (µg/L)	0.318	<0.04	1.391	0.057	1.257
	max (µg/L)	2.066	<0.04	1.932	0.231	4.673
	mean (µg/L)	1.114 ^a,b^	<0.04	1.643	0.058	4.098
SEM of mean		0.597		0.472	0.09	1.391
*p*-value		0.034		0.786	0.118	0.431

n (pos), percentage of positive samples (percentage of samples above LOD); α-ZEL, alpha-zearalenol; β-ZEL, beta-zearalenol; ZAN, zearalanone; α-ZAL, alpha-zearalanol; β-ZAL, beta-zearalanol; SEM—standard error of the difference between the means; min—the lowest detected concentration of ZEN metabolites in the sample; max—the highest detected concentration of ZEN metabolites in the sample; ^a,b^—different letters indicate significant differences between the means of averages in the same column (*p* < 0.05).

**Table 3:** Concentrations of ZEN metabolites in urine samples of dairy cows by stages.

		Zearalenone Metabolites				
		α-ZEL	α-ZAL	β-ZEL	β-ZAL	ZAN
1st stage (*n* = 10)	n (pos)	20	100	100	100	100
	min (µg/L)	0.177	0.216	0.217	0.155	0.129
	max (µg/L)	0.247	0.588	3.399	0.704	1.127
	mean (µg/L)	0.049	0.455	1.977	0.397 ^b^	0.602
2nd stage (*n* = 10)	n (pos)	40	100	100	100	100
	min (µg/L)	0.265	0.421	1.648	0.022	0.298
	max (µg/L)	0.354	0.873	5.158	0.297	1.988
	mean (µg/L)	0.124	0.611	3.1	0.207 ^a^	0.736
3rd stage (*n* = 10)	n (pos)	0	100	100	100	100
	min (µg/L)	<0.04	0.291	1.001	0.026	0.878
	max (µg/L)	<0.04	0.841	3.484	0.705	1.408
	mean (µg/L)	<0.04	0.594	2.753	0.343 ^b^	1.136
SEM of mean		0.077	0.151	0.675	0.047	0.451
*p*-value		0.184	0.317	0.172	0.005	0.055

n (pos), percentage of positive samples (percentage of samples above LOD); α-ZEL, alpha-zearalenol; β-ZEL, beta-zearalenol; ZAN, zearalanone; α-ZAL, alpha-zearalanol; β-ZAL, beta-zearalanol; SEM—standard error of the difference between the means; min—the lowest detected concentration of ZEN metabolites in the sample; max—the highest detected concentration of ZEN metabolites in the sample; ^a,b^—different letters indicate significant differences between the means of averages in the same column (*p* < 0.05).

**Table 4:** Pearson correlation coefficient between zearalenone and its metabolites in different biological fluids and feed.

		β-ZEL in Serum	β-ZAL in Serum	ZAN in Urine	β-ZAL in Urine	α-ZAL in Urine	ZEN in Feed
**α-ZEL in serum**	r	0.317	0.826 **	0.233	−0.305	−0.011	0.273
	*p-Value*	0.249	0.0001	0.403	0.270	0.970	0.477
**ZAN in** **serum**	r	0.570 *	0.377	0.518 *	−0.284	0.116	−0.605
	*p-Value*	0.027	0.166	0.048	0.305	0.680	0.084
**β-ZEL in urine**	r	−0.245	0.336	−0.125	−0.540 *	0.098	−0.360
	*p-Value*	0.379	0.220	0.657	0.038	0.727	0.342
**α-ZEL in urine**	r	0.330	0.394	−0.028	0.125	−0.064	−0.507
	*p-Value*	0.230	0.147	0.921	0.657	0.821	0.164
**ZEN in feed**	r	0.248	0.162	0.050	0.761 *	0.807 **	-
	*p-Value*	0.520	0.675	0.898	0.017	0.003	
**ZEN in milk**	r	-	-	-	-	-	0.411
	*p-Value*						0.271

α-ZEL, alpha-zearalenol; β-ZEL, beta-zearalenol; ZAN, zearalanone; β-ZAL, beta-zearalanol; r, correlation coefficient; * *p* < 0.05; ** *p* < 0.01.

## Data Availability

The data presented in this study are available within the article.

## References

[1] Hussein S.H., Jeffrey M.B. (2001). Toxicity, Metabolism, and Impact of Mycotoxins on Humans and Animals. Toxicology.

[2] Thapa A., Horgan K.A., White B., Walls D. (2021). Deoxynivalenol and Zearalenone—Synergistic or Antagonistic Agri-Food Chain Co-Contaminants?. Toxins.

[3] Murugesan G.R., Ledoux D.R., Naehrer K., Berthiller F., Applegate T.J., Grenier B., Phillips T.D., Schatzmayr G. (2015). Prevalence and effects of mycotoxins on poultry health and performance, and recent development in mycotoxin counteracting strategies. Poult. Sci..

[4] Pereira C.S., Cunha S.C., Fernandes J.O. (2019). Prevalent Mycotoxins in Animal Feed: Occurrence and Analytical Methods. Toxins.

[5] Gromadzka K., Waskiewicz A., Chełkowski J., Golinski P. (2008). Zearalenone and its metabolites: Occurrence, detection, toxicity and guidelines. World Mycotoxin J..

[6] Ben Salah-Abbès J., Belgacem H., Ezzdini K., Abdel-Wahhab M.A., Abbès S. (2020). Zearalenone nephrotoxicity: DNA fragmentation, apoptotic gene expression and oxidative stress protected by *Lactobacillus plantarum* MON03. Toxicon.

[7] Mahato D.K., Devi S., Pandhi S., Sharma B., Maurya K.K., Mishra S., Dhawan K., Selvakumar R., Kamle M., Mishra A.K. (2021). Occurrence, Impact on Agriculture, Human Health, and Management Strategies of Zearalenone in Food and Feed: A Review. Toxins.

[8] Busk Ø.L., Ndossi D., Verhaegen S., Connolly L., Eriksen G., Ropstad E., Sørlie M. (2011). Relative quantification of the proteomic changes associated with the mycotoxin zearalenone in the H295R steroidogenesis model. Toxicon.

[9] Kowalska K., Habrowska-Górczyńska D.E., Piastowska-Ciesielska A.W. (2016). Zearalenone as an endocrine disruptor in humans. Environ. Toxicol. Pharmacol..

[10] Bulgaru C.V., Marin D.E., Pistol G.C., Taranu I. (2021). Zearalenone and the Immune Response. Toxins.

[11] Metzler M., Pfeiffer E., Hildebrand A. (2010). Zearalenone and its metabolites as endocrine disrupting chemicals. World Mycotoxin J..

[12] Hort V., Nicolas M., Travel A., Jondreville C., Maleix C., Baéza E., Engel E., Guérin T. (2020). Carry-over assessment of fumonisins and zearalenone to poultry tissues after exposure of chickens to a contaminated diet-A study implementing stable-isotope dilution assay and UHPLC-MS/MS. Food Control.

[13] EFSA Panel on Contaminants in the Food Chain (2016). Scientific opinion on the appropriateness to set a group health-based guidance value for zearalenone and its modified forms. EFSA J..

[14] Liu J., Applegate T. (2020). Zearalenone (ZEN) in Livestock and Poultry: Dose, Toxicokinetics, Toxicity and Estrogenicity. Toxins.

[15] Barański W., Gajęcka M., Zielonka Ł., Mróz M., Onyszek E., Przybyłowicz K.E., Nowicki A., Babuchowski A., Gajęcki M.T. (2021). Occurrence of Zearalenone and Its Metabolites in the Blood of High-Yielding Dairy Cows at Selected Collection Sites in Various Disease States. Toxins.

[16] Muñoz-Solano B., González-Peñas E. (2020). Mycotoxin determination in animal feed: An LC-FLD method for simultaneous quantification of aflatoxins, ochratoxins and zearelanone in this matrix. Toxins.

[17] Paz H.A., Anderson C.L., Muller M.J., Kononoff P.J., Fernando S.C. (2016). Rumen Bacterial Community Composition in Holstein and Jersey Cows Is Different under Same Dietary Condition and Is Not Affected by Sampling Method. Front. Microbiol..

[18] Kachuei R., Rezaie S., Yadegari M.H., Safaie N., Allameh A.A., Aref-poor M.A., Fooladi A.A.I., Riazipour M. (2014). Determination of T-2 Mycotoxin in *Fusarium* strains by HPLC with fluorescence detector. Appl. Biotechnol. Rep..

[19] Rogowska A., Pomastowski P., Rafińska K., Railean-Plugaru V., Złoch M., Walczak J., Buszewski B. (2019). A study of zearalenone biosorption and metabolisation by prokaryotic and eukaryotic cells. Toxicon.

[20] Dänicke S., Winkler J. (2015). Invited review: Diagnosis of zearalenone (ZEN) exposure of farm animals and transfer of its residues into edible tissues (carry over). Food Chem. Toxicol..

[21] Dänicke S., Keese C., Meyer U., Starke A., Kinoshita A., Rehage J. (2014). Zearalenone (ZEN) metabolism and residue concentrations in physiological specimens of dairy cows exposed long-term to ZEN-contaminated diets differing in concentrate feed proportions. Arch. Anim. Nutr..

[22] Malekinejad H., Maas-Bakker R., Fink-Gremmels J. (2006). Species differences in the hepatic biotransformation of zearalenone. Vet. J..

[23] Zhang G.-L., Feng Y.-L., Song J.-L., Zhou X.-S. (2018). Zearalenone: A Mycotoxin With Different Toxic Effect in Domestic and Laboratory Animals’ Granulosa Cells. Front. Genet..

[24] EFSA CONTAM Panel (2017). Scientific opinion on risk for animal health related to the presence of zearalenone and its modified forms in feed. EFSA J..

[25] Oldenburg E., Forsten L. (1998). Mykotoxine im Grundfutter und ihre Bedeutung im Carry-over-Geschehen. Kreisläufe Erwün-Schter und Unerwünschter Stoffe–Ihre Bedeutung in der Nahrungskette, Informationsveranstaltung der Arbeitsgruppe “Carry Over Unerwünschter Stoffe in Futtermitteln”, Braunschweig, Germany, October 1998.

[26] Huang L.C., Zheng N., Zheng B.Q., Wen F., Cheng J.B., Han R.W., Xu X.M., Li S.L., Wang J.Q. (2014). Simultaneous determination of aflatoxin M_1_, ochratoxin A, zearalenone and α-zearalenol in milk by UHPLC–MS/MS. Food Chem..

[27] Seeling K., Dänicke S., Ueberschär K.H., Lebzien P., Flachowsky G. (2005). On the effects of *Fusarium* toxin-contaminated wheat and the feed intake level on the metabolism and carry over of zearalenone in dairy cows. Food Addit. Contam..

